# C16-ceramide modulates sex-dimorphic defense strategies under cold stress in *Cherax quadricarinatus* via JAK-STAT suppression and oxidative disruption

**DOI:** 10.1186/s13293-026-00892-y

**Published:** 2026-03-26

**Authors:** Honglin Chen, Huiyi Cai, Shuzhi Li, Xiaojun Xu, Bao Lou

**Affiliations:** 1 State Key Laboratory for Managing Biotic and Chemical Threats to the Quality and Safety of Agro-products, Institute of Hydrobiology, Zhejiang Academy of Agricultural Sciences, Hangzhou, 310021 China; 2 College of Life Sciences, China Jiliang University, Hangzhou, 310018 China

**Keywords:** Cold stress, Sexual dimorphism, JAK-STAT signaling pathway, C16-ceramide

## Abstract

**Background:**

Extreme low temperatures impose severe physiological stress on aquatic crustaceans. Understanding the molecular mechanisms of cold adaptation is critical for improving stress resilience in key aquaculture species. This study employed integrated transcriptomic and metabolomic analyses to decipher sex-divergent neuroendocrine strategies in *Cherax quadricarinatus* under cold stress.

**Results:**

Males rely on high basal antioxidant capacity for rapid JAK-STAT pathway activation to combat acute cold stress, whereas females maintain metabolic homeostasis through sustained pathway activation. This sexually dimorphic strategy is negatively regulated by C16-ceramide. Females relied on sustained JAK-STAT activation for transcriptional reprogramming, while males exhibited rapid metabolic remodeling and transient antioxidant mobilization. Crucially, the sphingolipid hub molecule C16-ceramide was coordinately downregulated in both sexes. Exogenous supplementation revealed C16-ceramide potently suppressed JAK-STAT genes and disrupted hepatopancreatic redox homeostasis in females, while triggering antioxidant collapse in males.

**Conclusions:**

This study provides the first evidence of sexual dimorphism in JAK-STAT mediated cold adaptation in invertebrates, identifies C16-ceramide as a regulator of sex-specific defense strategies, and confirms the penetration of sexual dimorphism into conserved molecular stress response networks.

**Supplementary Information:**

The online version contains supplementary material available at 10.1186/s13293-026-00892-y.

## Highlights

1. First evidence of sexual dimorphism in invertebrate cold adaptation via JAK-STAT pathway.
2. Identifies sphingolipid (C16-Ceramide) as a critical cold adaptation regulator.
3. Integrated omics uncovers molecular basis for differential climate resilience with aquaculture implications.

**Supplementary Information:**

The online version contains supplementary material available at 10.1186/s13293-026-00892-y.

## Abstract (plain language summary)

When water get extremely cold, animals like crayfish experience dangerous stress just like humans do during freezing weather. Interestingly, we discovered that male and female red claw crayfish (*Cherax quadricarinatus*) which is an important farmed species, handle this cold stress in completely different ways. Males act like quick responders, they immediately trigger their stress alarm JAK-STAT system and use their natural antioxidant shields to survive sudden chills. Females take a steadier approach, they keep this alarm system constantly active to carefully balance their inner body chemistry during prolonged cold exposure. The biggest surprise was finding that a tiny fat molecule, C16-ceramide, works like a biological switch controlling these sex-specific survival tactics. Normally, both males and females reduce this molecule when cold. But after added extra amounts, it broke the stress-response systems causing cellular damage in females, while destroyed their protective antioxidant shields in males. This is the first time such fundamental male-female stress response differences have been found in spineless animals, these findings help crayfish farmers better protect their stocks during cold snaps by tailoring treatments differently for males and females, and reveal how biological sex shapes life or death responses at the most basic cellular level which might apply to all animals.

**Supplementary Information:**

The online version contains supplementary material available at 10.1186/s13293-026-00892-y.

## Introduction

Water temperature is a critical environmental factor influencing the biology and physiology of aquatic animals [1, 2]. For introduced species, climatic variations in new farming areas, particularly temperature regimes, can induce stress that negatively impacts production, disease outbreaks and reproduction performance [3, 4]. For example, cold stress compromises growth performance and immunity in *Cherax quadricarinatus* [5], while acute heat stress impairs antioxidant status and immune function in the intestine of *Procambarus clarkii* [6]. Crucially, the neuroendocrine system within the X Organ-Sinus Gland (XO-SG) complex plays a vital role in maintaining homeostasis under stress conditions in crustaceans [7–10]. This is evidenced by heat and cold shock inducing hemolymph norepinephrine release in *Macrobrachium rosenbergii* [11]and salinity stress altering multiple neuropeptide families originating from the XO-SG complex in *Carcinus maenas* [12].

Crustaceans exhibit significant sexual dimorphism across multiple biological scales. Morphologically, males often develop enlarged chelae for intraspecific competition [13–15], while females possess specialized abdominal structures for egg incubation [16, 17]. Physiologically, females incur greater metabolic costs due to vitellogenin synthesis [18, 19], whereas males maintain higher basal metabolic rates to support activity demands [20, 21], reflecting divergent metabolic priorities that females favor energy storage and males prioritize locomotion [22, 23]. Sex control techniques are established in commercially important decapod crustaceans and recognized for improving aquaculture efficiency [24, 25]. However, while cold-response mechanisms in crustaceans, such as heat shock protein (HSP) regulation and antifreeze glycoprotein accumulation, are identified [26–28], the influence of sex on these responses is often overlooked despite documented physiological differences in metabolic efficiency, antioxidant capacity, and behavior between sexes [29, 30].

Understanding temperature response mechanisms in aquatic organisms is crucial for enhancing adaptation in introduced species, necessitating focused research on neuroendocrine regulation. Current knowledge primarily addresses hepatopancreatic responses to cold stress [31–33], while the role of the eyestalk, a major neuroendocrine center, remains less explored. This study employed *Cherax quadricarinatus*, a commercially important introduced aquatic species in China, as a model and integrated transcriptomic and metabolomic analyses of eyestalk tissue with dietary supplementation of exogenous C16-ceramide to investigate sexually dimorphic responses to cold stress. Multi-omics data revealed distinct sex-specific strategies. Both sexes exhibited downregulation of sphingolipid signaling under cold stress. Crucially, C16-ceramide supplementation under cold stress disrupted JAK-STAT signaling networks in females, while impairing antioxidant defenses in males. These findings elucidate mechanisms underlying sexual dimorphism in invertebrate cold stress adaptation and provide new insight into the evolutionary significance of sex-specific stress responses in crustaceans.

## Methods

### Animals and experimental set up

*C. quadricarinatus* were obtained from the Breeding Center, Zhejiang Academy of Agricultural Sciences, China. Forty-four adult crayfish (22 females and 22 males) were equally divided into two groups. The cold-stress group (CS) experienced gradual temperature reduction from 27 °C to 15 °C at 1 °C / 0.5 h, followed by 72 h maintenance at 15 °C. Crayfish maintained at 27 °C served as controls (CT). Eyestalks were collected from both groups for transcriptomic and metabolomic analyses post-treatment.

### RNA-seq analysis

A total of 20 samples (2 sexes × 2 temperature stress treatments × 5 biological replications) were prepared for transcriptome sequencing. Total RNA was extracted from eyestalk tissue of *C. quadricarinatus* using the RNeasy Mini Kit (Qiagen, German), following the instructions. The quality and quantity of RNA were assessed by agarose gel electrophoresis and a NanoDrop 2000 spectrophotometer (ThermoFisher Scientific, USA). A total of 20 libraries were constructed using the NEBNext Ultra RNA Library Prep Kit (Illumina), followed by sequencing on the NovaSeq X Plus platform (Illumina, CA, USA). The raw data of RNA-seq reads were filtered and the clean data were mapped to the reference genome of *C. quadricarinatus* [34] using hisat2 (http://ccb.jhu.edu/software/hisat2/index.shtml). FPKMs of each sample were calculated by RSEM (http://deweylab.github.io/RSEM/).

### LC-MS/MS analysis

A total of 24 eyestalk samples (2 sexes × 2 temperature stress treatments × 6 biological replications) were used for metabolomics analysis. The metabolites of each sample were extracted using 400 µL methanol: water (4:1, v / v) solution with 0.02 mg / mL L-2-chlorophenylalanin as internal standard. A pooled in house quality control sample (QC) was prepared by mixing equal volumes of all samples. The LC-MS/MS analysis of sample was conducted on a UHPLC-Q Exactive HF-X system. Raw data of LC/MS is preprocessed by Progenesis QI (Waters Corporation, Milford, USA) software. The metabolites were searched and identified, and the main database was the HMDB (http://www.hmdb.ca/)、Metlin ( https://metlin.scripps.edu/) and Majorbio Database. Metabolic features detected at least 80% in any set of samples were retained. Log10 logarithmization was performed to obtain the final data matrix for subsequent analysis. The subsequently data analysis such as partial least squares discriminant analysis (PLS-DA), screening of differentially expressed genes (DEGs) and metabolites (DEMs), VIP validation, as well as KEGG enrichment and visualization were carried out on the online platform of majorbio cloud platform (https://cloud.majorbio.com).

### Transcriptome validation

Validation of transcriptomic data was performed via qRT-PCR analysis on six genes (*ALF*, *HSP70*, *VOM1*, *Trx*, *COX2*, *CAT*) using *18 S rRNA* as the reference gene (Table. S1). Total RNA (1 µg) was reverse-transcribed with Hifair^®^ III 1st Strand cDNA Synthesis SuperMix (Yeasen, China). Reactions were conducted in 5 biological replicates and 3 technical replicates. with Hieff^®^ qPCR SYBR Green Master Mix (Yeasen, China) under standardized cycling conditions: 95 °C for 3 min; 40 cycles of 95 °C for 10 s, 60 °C for 20 s, and 72 °C for 20 s. Relative expression values were obtained using the 2^−∆∆Ct^ method.

### Dynamic expression profiling of JAK-STAT components

Based on transcriptomic profiling, core components of the JAK-STAT pathway (*Domeless*, *VEGFR*, *STAT2*, *STAT5B*, *Bcl-xL*, *PIM1*, *CIS*, *SOCS*, *PIAS*) were selected for dynamic expression analysis. 50 female and 50 male crayfish were acclimated for one week before cold stress. In contrast with the moderate stress regimen used for global transcriptomic and metabolomic profiling, a more intense cold stress protocol (from 27 °C to 9 °C) was applied to elucidate the dynamics expression and inflection point of the JAK-STAT pathway. Starting at 27 °C, temperature was reduced by 1 °C per 4 h until reaching 9 °C at 72 h, then maintained at 9 °C for 5 days. Eyestalk tissues were collected at 0 h, 4 h, 8 h, 12 h, 24 h, 36 h, 48 h, 60 h, and 72 h of cold stress, and after 5 d at 9 °C. Samples were flash-frozen in liquid nitrogen and stored at -80 °C, with five biological replicates per time point. qRT-PCR was performed as described above. Primer sequences for all target genes and the *18* *S* *rRNA* reference gene are listed in Table 1.

**Table 1:** Primer sequences used for expression profiling of JAK-STAT components in *C. quadricarinatus*

Gene name	Primer name	Sequences (5’-3’)
*Domeless*	Forward	TCAGAGTGGTCTCCCTGCTT
	Reverse	GCTACTGCAACGTACCCTGT
*VEGFR*	Forward	TGACCGAGGCTGATTCACAA
	Reverse	CGCCCTTCTTCCACTTTGGT
*STAT5B*	Forward	AGGAGTGGTGTGAGGCACTA
	Reverse	AAGACAAGAGGCGGGTGATG
*STAT2*	Forward	GCTGCACATGAAGTGGTTGG
	Reverse	CTGGAACAGAGGGTGTGGAC
*Bcl-xL*	Forward	ACATCACCAGAGAGACGGGA
	Reverse	AGTGCCAACTGTCCACCAAA
*PIM1*	Forward	TCCCTGGCTCAACAACACTC
	Reverse	CTCTTGGGACGAGGTGGAAC
*CIS*	Forward	CCAAGGAAACCGACAGCG
	Reverse	AGCGGAATCCCGTAGCAG
*SOCS*	Forward	GGAAAGTGTCGGCAGCAAAG
	Reverse	CTATTGGGCTGCGGTGAAGA
*PIAS*	Forward	CTACACCCATCCCCACCAAC
	Reverse	CGACCATATTCCGAAGCCCA
*18 S rRNA*	Forward	CTGAGAAACGGCTACCACATC
	Reverse	GCCGGGAGTGGGTAATTT

### C16-ceramide diet preparation and feeding trial

Based on transcriptomic profiling, an experimental diet was formulated by supplementing the basal diet (36% crude protein and 3% crude fat) with 50 mg/kg C16-ceramide dissolved in 0.1 mL ethanol and homogenized via stepwise mixing, with 0.02% vitamin E added to prevent lipid peroxidation [35]. Ingredients were pulverized, sieved (90-mesh), mixed with double-distilled water, extruded into 2-mm pellets, dried at 45 °C for 4 h, and stored at -20 °C. Three experimental groups (*n* = 20 per group: 10 females + 10 males) comprised: cold-stress basal diet (CS-BD), cold-stress C16-ceramide diet (CS-C16), and ambient-temperature basal diet (CT-BD, 27 °C). Cold stress was induced one week after feeding experiment with temperature reduction from 27 °C at 1 °C/4 h. Based on sex-specific response peaks identified in JAK-STAT dynamic expression analysis, eyestalks and hepatopancreas were collected from females at 48 h and males at 24 h post-stress initiation, followed by immediate flash-freezing in liquid nitrogen and light-protected storage at -80 °C.

### Biochemical enzyme and antioxidant enzyme activity measurement

Hepatopancreas samples were processed by homogenizing in PBS at a ratio of 1:9 (0.1 mg:0.9 µl), followed by centrifugation at 3000 rpm for 10 min at 4℃, and the supernatant was then collected for further biochemical enzyme and antioxidant enzyme activity measurement. The alanine aminotransferase (ALT) (C009-2-1, Nanjing Jiancheng) and aspartate transaminase (AST) (C010-2-1, Nanjing Jiancheng), total activities of superoxide dismutase (T-SOD) (A001-3-2, Nanjing Jiancheng), malondialdehyde (MDA) (A003-1-2, Nanjing Jiancheng), catalase (CAT) (A007-1-1, Nanjing Jiancheng), and glutathione peroxidase (GSH-Px) (A005-1-2, Nanjing Jiancheng) were measured using commercially available ELISA kits (Nanjing Jiancheng Bioengineering Institute Co., Ltd., China) according to the manufacturer’s instructions.

### Statistical analysis

All the assays were carried out with at least five biological replicates. The analysis data are presented as mean ± SD. One-way analysis of variance (ANOVA) and Duncan’s tests were used to determine statistical significance (*P* < 0.05) between control and treatment groups. All statistical analyses were performed using Agricolae v1.3.7 R package.

## Results

### Overview of RNA-seq and metabolomic data quality

**Fig. 1:**
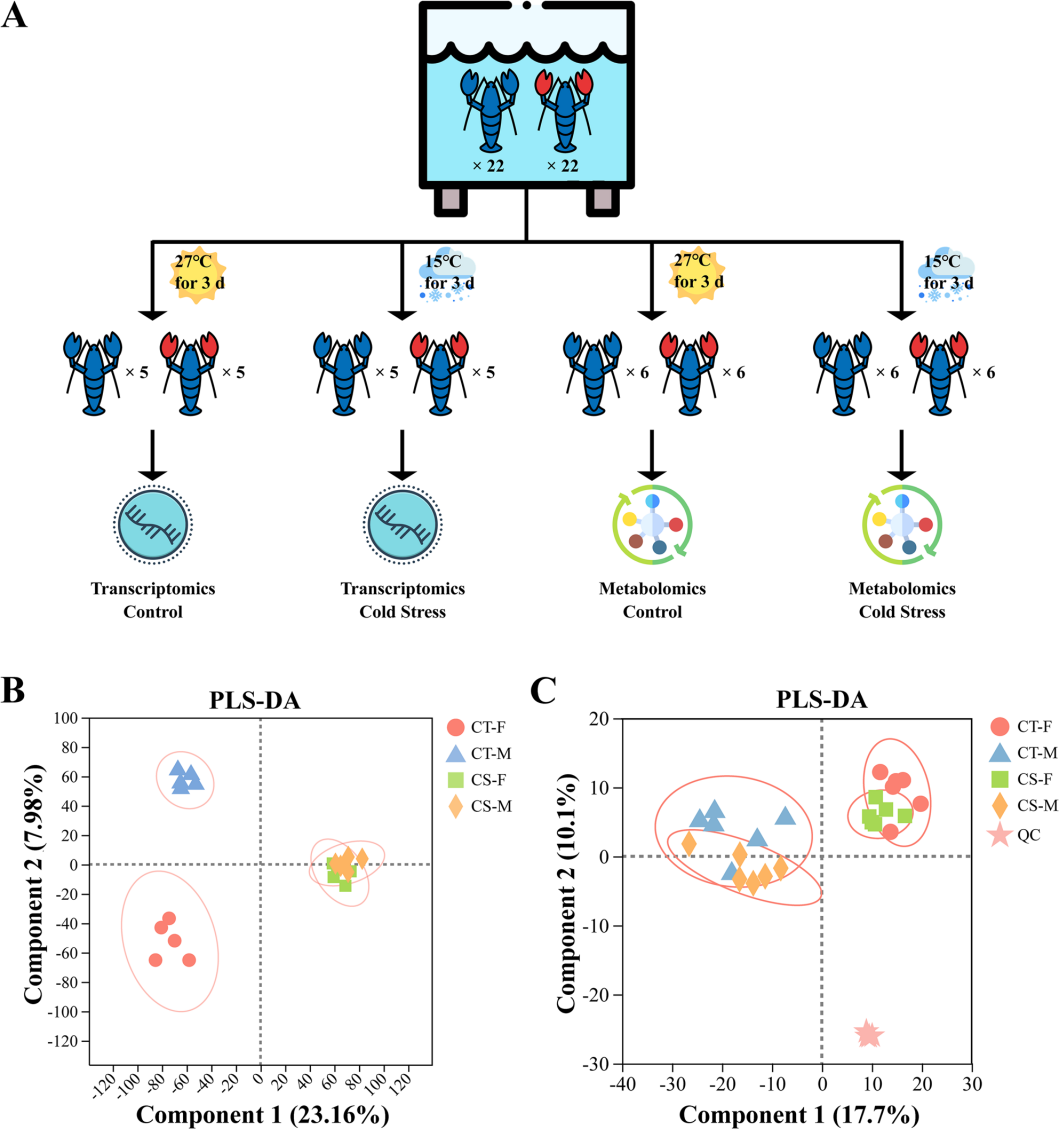
Multi-omics analysis of cold stress responses in *C. quadricarinatus. *(**A**) Schematic of cold stress treatment and sampling protocol for transcriptomic (*N* = 5) and metabolomics (*N* = 6) profiling. (**B-C**) PLS-DA score plots visualizing group separation in transcriptome. (**B**) and metabolome (**C**) datasets. Groups are represented as: CT-F (Female control individuals, red circles), CT-M (Male control individuals, blue triangles), CS-F (Female cold stress individuals, green squares), CS-M (Male cold stress individuals, orange rhombuses), and QC (Quality control samples, pink stars, *n* = 4). Ellipses denote 95% confidence intervals for group clusters

The transcriptomic analysis of 20 samples (Fig. 1A) generated a total of 138.80 Gb of clean data, with each sample yielding ≥ 6.04 Gb. The Q30 base percentage exceeded 95.31% across all samples. The clean reads from each sample were aligned to the designated reference genome (10.1038/s41597-023-02124-zIF: 6.9 Q1 B2), with alignment rates ranging from 89.65% to 90.15% (Table S2). qRT-PCR validation confirmed transcriptomic profiles for all six candidate genes (*ALF*, *HSP70*, *VOM1*, *Trx*, *COX2*, *CAT*), with expression trends consistent with RNA-seq data. The Pearson’s correlation coefficient (R-value) of RT- PCR and RNA-seq was 0.97 and 0.95, which showed a high consistency of the RT-PCR and RNA-seq data (Figure S1).

A total of 6,064 ion peaks were detected across 24 metabolomic samples (Fig. 1A), with 937 successfully annotated to metabolites (619 in positive ion mode, 318 in negative ion mode). Quality control (QC) assessments demonstrated robust stability, with a relative standard deviation (RSD) < 0.3 and 81.81% cumulative proportion of qualified peaks, confirming acceptable data quality.

### PLS-DA of the transcriptomic and metabolic data

Partial least squares discriminant analysis (PLS-DA) of the transcriptomic data revealed distinct clustering patterns driven by cold stress treatment (Fig. 1B). The first two components explained 31.14% of the total variance. Along comp1, samples segregated primarily by temperature, while along comp2, the samples in CT groups separated by sexes, and there was no significant separation in cold stress group. The distinct clustering patterns were observed between CS and CT groups, demonstrating that temperature stress significantly reshaped the global gene expression profiles of eyestalk tissues. PLS-DA of the metabolic data showed QC samples (*N* = 4) formed a tight group, confirming technical robustness (Fig. 1C). Compared to transcriptomics, metabolic variance was more influenced by sex. The first two components collectively accounted for 27.80% of the total variance. Both CT groups and CS groups exhibited clear sexual dimorphism, reflecting inherent metabolic divergence between sexes. Both female and male specimens displayed overlapping metabolic profiles between CS and CT groups, with diffuse clustering patterns. These results highlight the need for sex-specific analysis in stress studies.

### DEGs and DEMs screening and KEGG enrichment

**Fig. 2:**
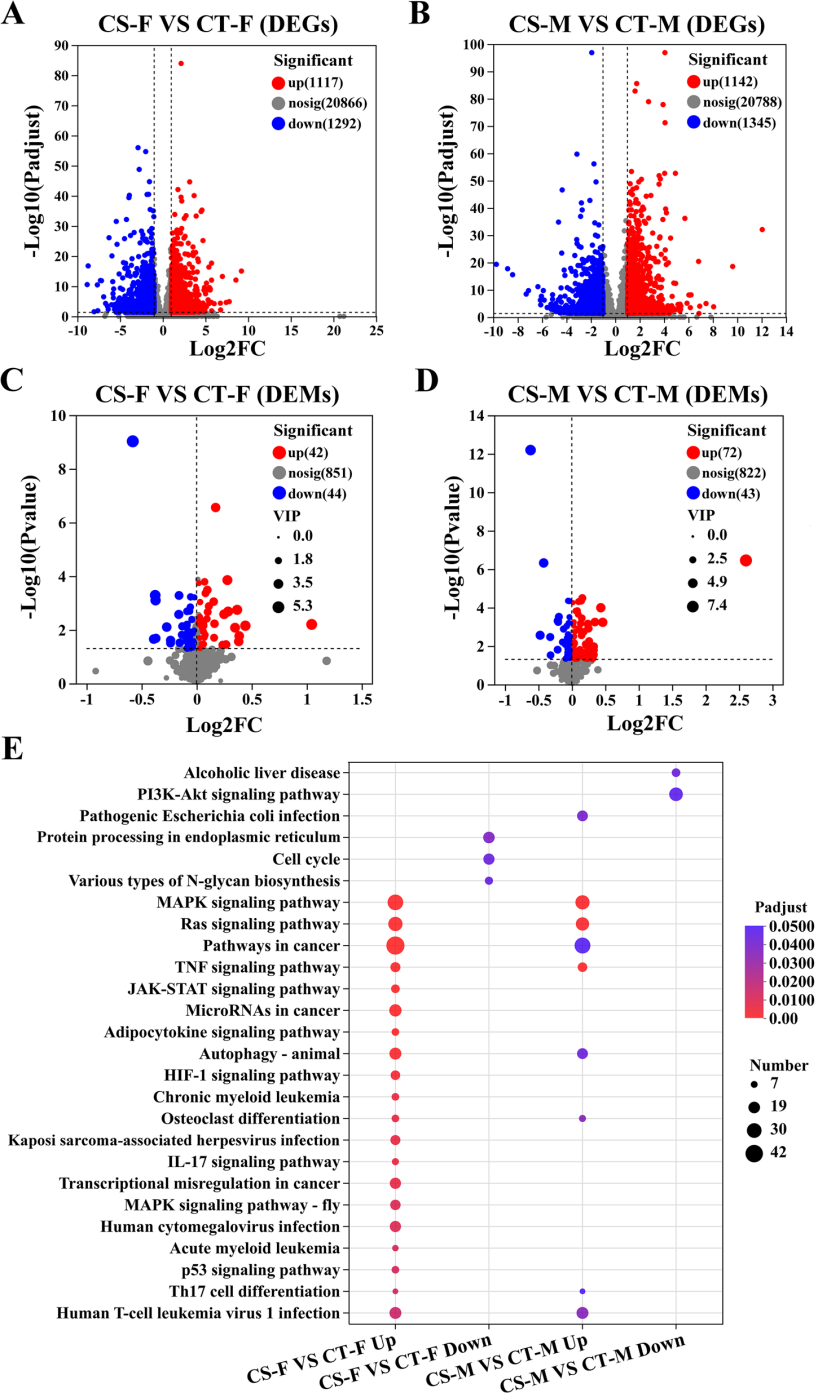
Transcriptomic and metabolomic signatures of cold stress responses. (**A-D**) Volcano plots of differentially expressed genes (DEGs) and metabolites (DEMs) in cold-stressed (CS) vs. control (CT) groups. (**A**) DEGs in females (CS-F vs. CT-F), (**B**) DEGs in males (CS-M vs. CT-M), (**C**) DEMs in females (CS-F vs. CT-F), (D) DEMs in males (CS-M vs. CT-M). Red points significantly upregulated in CS (*P*-adjust < 0.05, log2FC ≥ 1 for DEGs; *P*-value < 0.05, VIP > 1 for DEMs), blue points significantly downregulated in CS (*P*-adjust < 0.05, log2FC ≤ -1 for DEGs; *P*-value < 0.05, VIP < 1 for DEMs). Gray points: Non-significant features. (**E**) KEGG pathway enrichment of top 20 significant terms for DEGs in CS vs. CT comparisons

Differentially expressed genes (DEGs) and metabolites (DEMs) were screened between CS and CT groups for both sexes (Fig. 2A-D). Female crayfish exhibited 2,409 DEGs (1,117 up regulated and 1,292 down regulated) and 86 DEMs (42 up regulated and 44 down regulated). In males, 2,487 DEGs (1,142 upregulated and 1,345 down regulated) and 115 DEMs (72 up regulated and 43 down regulated) were identified (Table S3, S4).

KEGG analysis revealed sexually dimorphic metabolic reprogramming in *C. quadricarinatus* under cold stress. Compared to the male, female crayfish exhibited broader transcriptomic activity, and uniquely engaging JAK-STAT signaling, HIF-1-mediated hypoxia adaptation, and p53-dependent genomic surveillance, coupled with adipocytokine-mediated metabolic regulation and IL-17-driven immune modulation (Fig. 2E). Sex-specific downregulation of PI3K-Akt signaling in male and endoplasmic reticulum protein processing in female highlighted divergent metabolic trade-offs (Table S5).

Metabolomic profiling showed male-specific activation of branched-chain amino acid (BCAA) biosynthesis/degradation and nucleotide metabolism under cold stress (Figure S2), potentially generating antioxidative BCAA derivatives and ATP to mitigate oxidative damage. Concomitant upregulation of ABC transporters suggested enhanced transmembrane homeostasis. Conversely, the absence of upregulated metabolic pathways in cold-stressed females indicated preferential reliance on transcriptional strategies through JAK-STAT signaling. Both sexes exhibited coordinated downregulation of energy-intensive processes including oxidative phosphorylation, sphingolipid signaling, and insulin resistance pathways (Table S6).

### Temperature gradient dynamics of JAK-STAT signaling

**Fig. 3:**
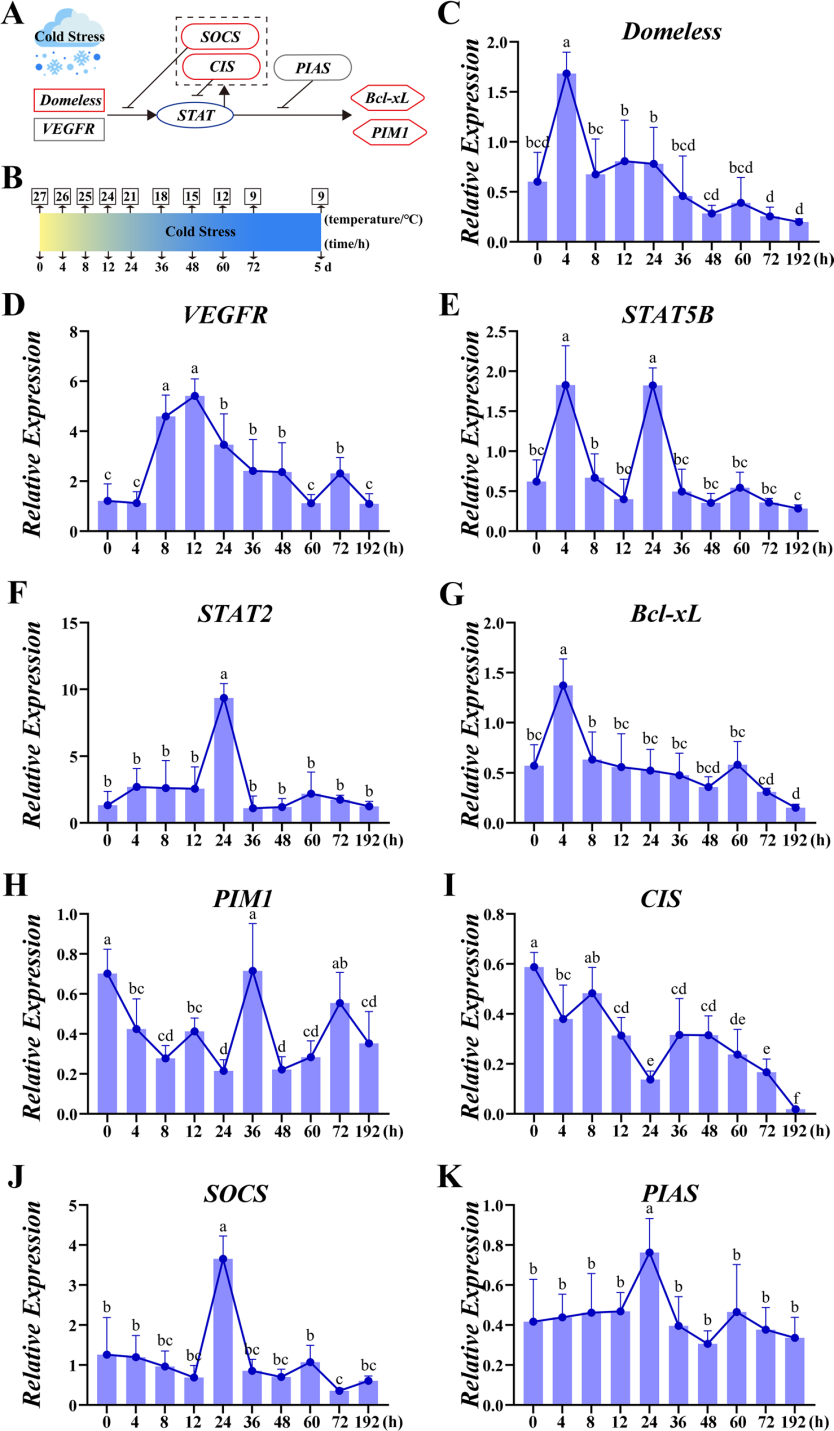
Male-specific JAK-STAT pathway dynamics in *Cherax quadricarinatus* under cold stress. (**A**) Schematic representation of the JAK-STAT signaling pathway with transcriptomic data integration (Genes significantly upregulated are indicated by red boxes, downregulated genes are indicated by blue boxes). (**B**) Timeline of cold stress experiment and sampling time points (0 h, 4 h, 8 h, 12 h, 24 h, 36 h, 48 h, 60 h, 72 h, and 192 h). (C-K) Expression profiles of key components including *Domeless* (**C**), *VEGFR* (**D**), *STAT5B* (**E**), *STAT2* (**F**), *Bcl*-*xL* (**G**), *PIM1* (**H**), *CIS* (**I**), *SOCS* (**J**), and *PIAS* (**K**) of JAK-STAT pathway across ten sampling intervals. Data represent mean ± SD (*N* = 5, per group)

**Fig. 4:**
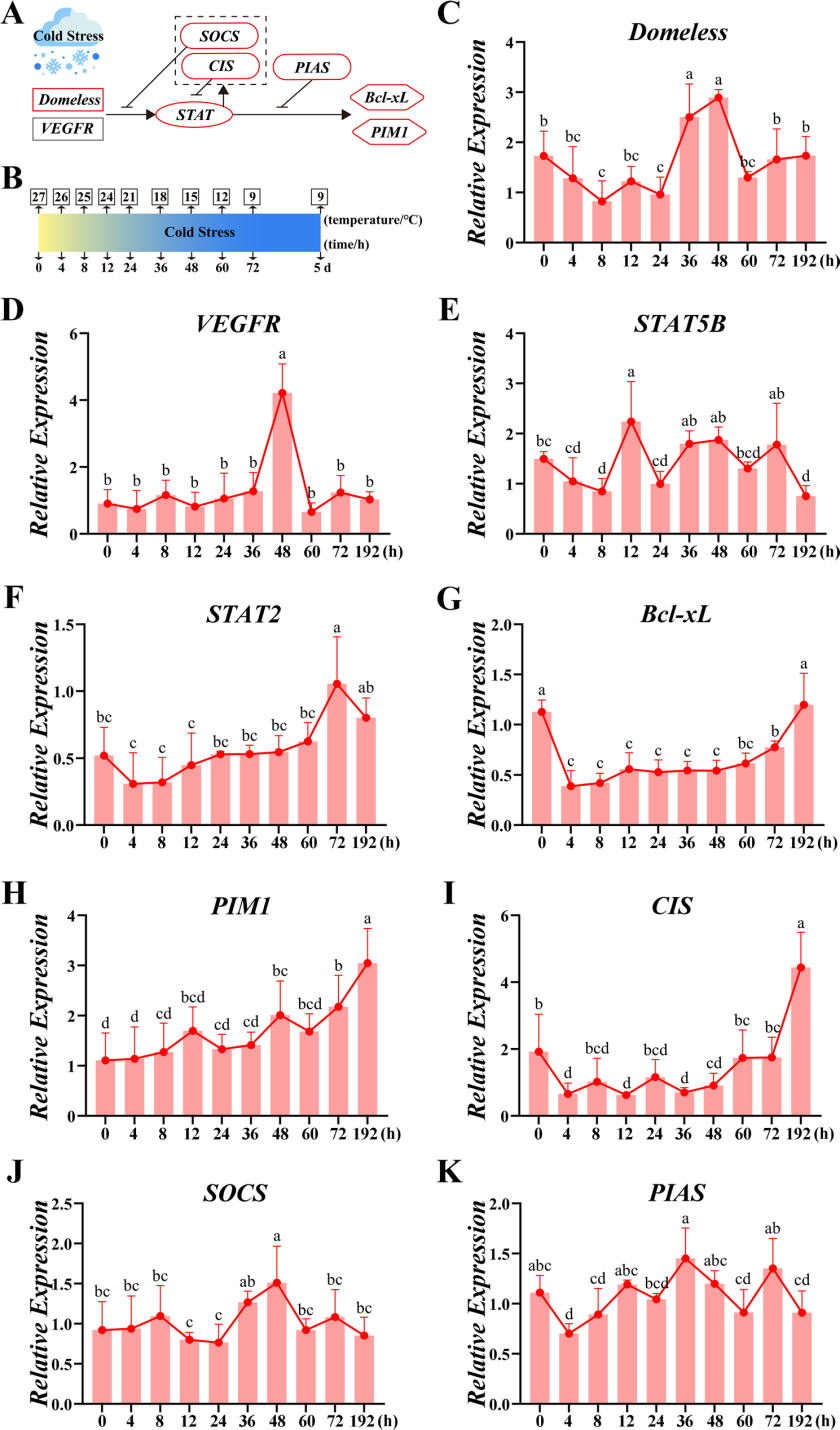
Female-specific JAK-STAT pathway dynamics in *C. quadricarinatus* under cold stress. (**A**) Schematic representation of the JAK-STAT signaling pathway with transcriptomic data integration (Genes significantly upregulated are indicated by red boxes). (**B**) Timeline of cold stress experiment and sampling time points (0 h, 4 h, 8 h, 12 h, 24 h, 36 h, 48 h, 60 h, 72 h, and 192 h). (**C-K**) Expression profiles of key components including *Domeless* (**C**), *VEGFR* (**D**), *STAT5B* (**E**), *STAT2* (**F**), *Bcl-xL* (**G**), *PIM1* (**H**), *CIS* (**I**), *SOCS* (**J**), and *PIAS* (**K**) of JAK-STAT pathway across ten sampling intervals. Data represent mean ± SD (*N* = 6 per group)

Comprehensive temporal profiling of JAK-STAT pathway components in *C. quadricarinatus* revealed profoundly divergent sex-specific kinetics during 192 h cold exposure (Figs. 3 and 4). Males exhibited immediate early-phase responses, characterized by rapid receptor activation, *Domeless* peaked at 4 h (Fig. 3C) and *VEGFR* maxima at 8–12 h (Fig. 3D). Transcriptional regulators showed fluctuating patterns as *STAT5B* displayed dual peaks (4 h and 24 h), while *STAT2* surged at 24 h (Fig. 3E and F). Negative regulators *SOCS* and *PIAS* synchronously peaked at 24 h (Fig. 3J and K), strikingly, another negative regulators *CIS* expression was dropped to its lowest point at 24 h (Fig. 3I). Downstream effector genes including anti-apoptotic *Bcl-xL* transiently spiked at 4 h, whereas proliferation kinase *PIM1* demonstrated triphasic peaks (0 h, 36 h, 72 h) (Fig. 3G and H).

Females manifested delayed response, with receptor *Domeless* at 36–48 h and *VEGFR* at 48 h (Fig. 4C). Transcriptional regulators displayed a slow rising trend, STAT5B expression fluctuated significantly, showing distinct peaks at 12 h, 36–48 h, and 72 h, interspersed with declines, and *STAT2* gradually ascended to maximum at 72 h (Fig. 4E and F). Negative feedback was fluctuant with *SOCS* peaked at 48 h and *PIAS* peaked at 36 h and 72 h (Fig. 4J and K). The *CIS* increased slowly and achieved its peak until 5d (Fig. 4I). *Bcl-xL* and *PIM1* both progressively increased to maxima along exposure time (Fig. 4G and H).

### Sex divergent effects of C16-ceramide supplementation

**Fig. 5:**
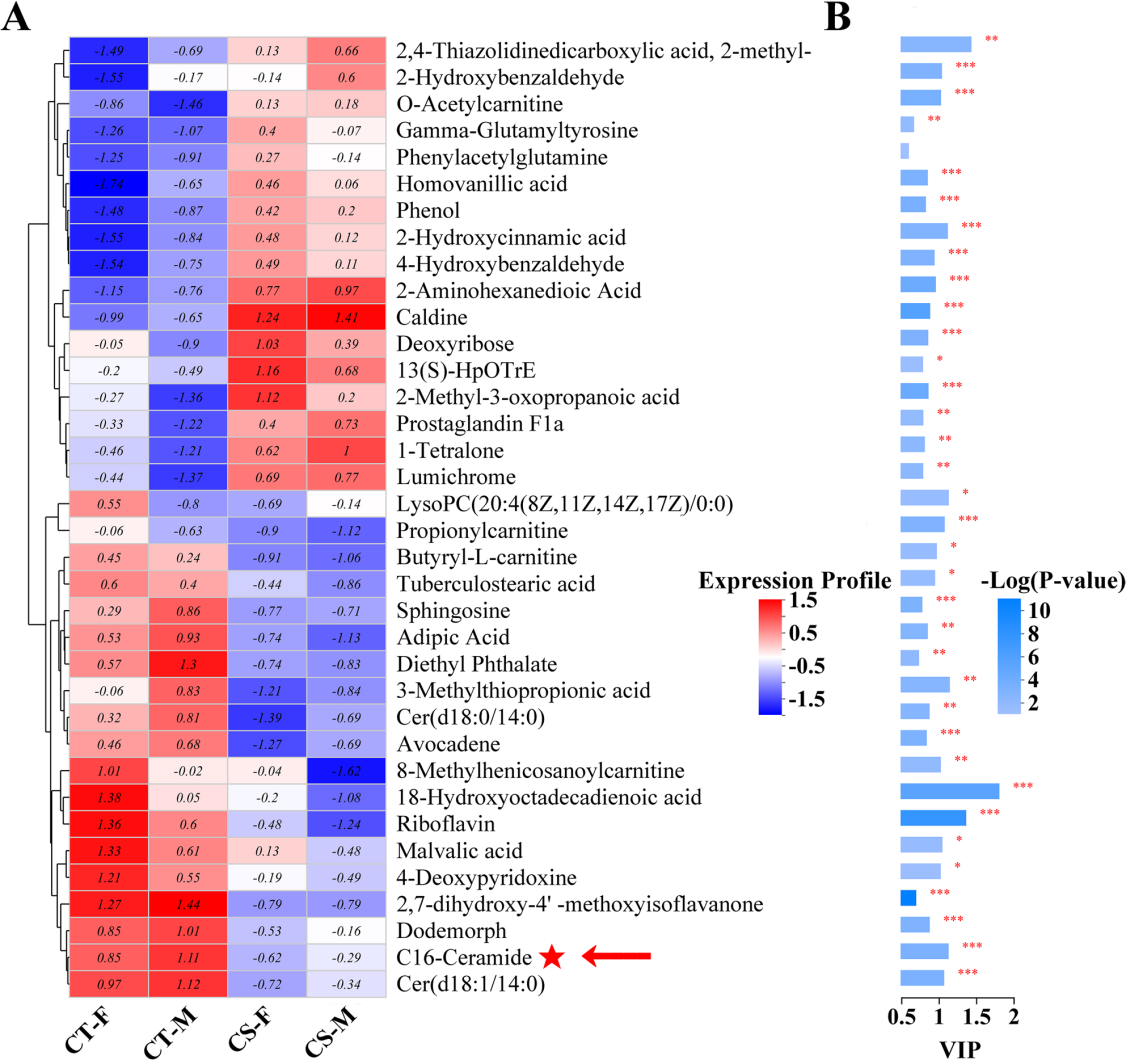
Expression profiles and variable importance (VIP) of metabolites. **A** Hierarchical clustering heatmap, rows represent individual metabolites, and columns represent sample group. The dendrogram illustrates the similarity of expression patterns among metabolites. The color intensity within each cell represents the relative abundance of a metabolite. **A** key differentially regulated metabolite C16-ceramide is indicated by a red arrow and asterisk. **B** Variable importance in projection (VIP) scores. The horizontal bar length indicates the VIP score for each metabolite. VIP scores are shown for metabolites meeting the threshold of ≥ 1.0. Bar color represents the statistical significance of the abundance of metabolite, corresponding to the -log₁₀ (*P*-value) scale. Significance levels are denoted by asterisks( * *P* < 0.05, ** *P* < 0.01, *** *P* < 0.001)

A total of 36 metabolites were identified that showed significant upregulation or downregulation in both females and males after exposure to cold stress, the expression profiles and variable importance (VIP) of these metabolites were visualized in Fig. 5. Among these metabolites, C16-ceramide was the key member of sphingolipid metabolism, and also involved in metabolic pathways, neurotrophin signaling pathway, adipocytokine signaling pathway, insulin resistance, necroptosis, sphingolipid signaling pathway.

**Fig. 6:**
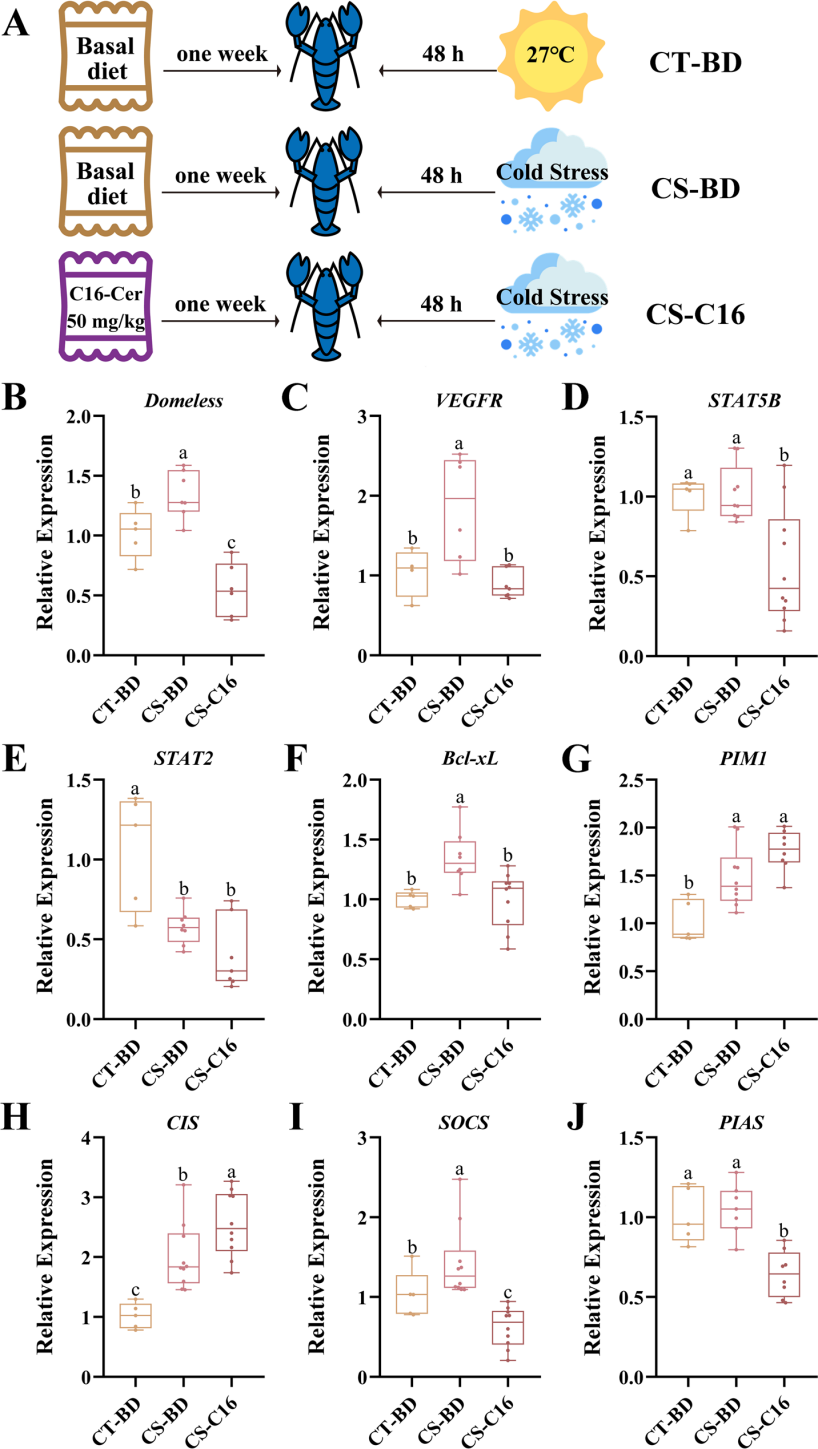
Modulation of JAK-STAT pathway by dietary C16-ceramide under cold stress in female *C. quadricarinatus*. (**A**) C16-ceramide induces comprehensive pathway suppression in female *C. quadricarinatus*. The expression of *VEGFR* (**D**), *STAT5B* (**E**), *STAT2* (**F**), *Bcl-xL* (**G**), *PIM1* (**H**), *CIS* (**I**), *SOCS* (**J**), and *PIAS* (**K**) of JAK-STAT pathway were analyzed across three experimental groups: ambient-temperature (27 °C) with basal diet (CT-BD), cold-stress with basal diet (CS-BD), and cold-stress with C16-ceramide diet (CS-C16). Data presented as mean ± SD (*N* = 6 per group)

To investigate the role of C16-ceramide in modulating cold stress responses, we supplemented basal diet with C16-ceramide and analyzed key components of the JAK-STAT pathway in male and female *C. quadricarinatus* at their respective response time points, females at 48 h and males at 24 h (Fig. 6A). In females, C16-ceramide induced comprehensive pathway suppression. The core receptor *Domeless* exhibited significantly lower expression in CS-C16 than both CT-BD and CS-BD groups (Fig. 6B). Similarly, *VEGFR* expression in CS-C16 group was suppressed, indicating impaired signal initiation (Fig. 6C). Downstream Transcriptional regulators mirrored this pattern, *STAT5B* decreased significantly in CS-C16 group, while *STAT2* remained suppressed in CS-C16 as CS-BD group (Fig. 6D and E). The anti-apoptotic *Bcl-xL* decreased after C16-ceramide supplement, though pro-survival *PIM1* maintained cold-induced levels (Fig. 6F and G). Negative regulators showed divergent responses, *PIAS* and *SOCS* were inhibited in CS-C16 compare to CS-BD group, while *CIS* expression was amplified, suggesting compensatory feedback activation (Fig. 6H, I and J).

**Fig. 7:**
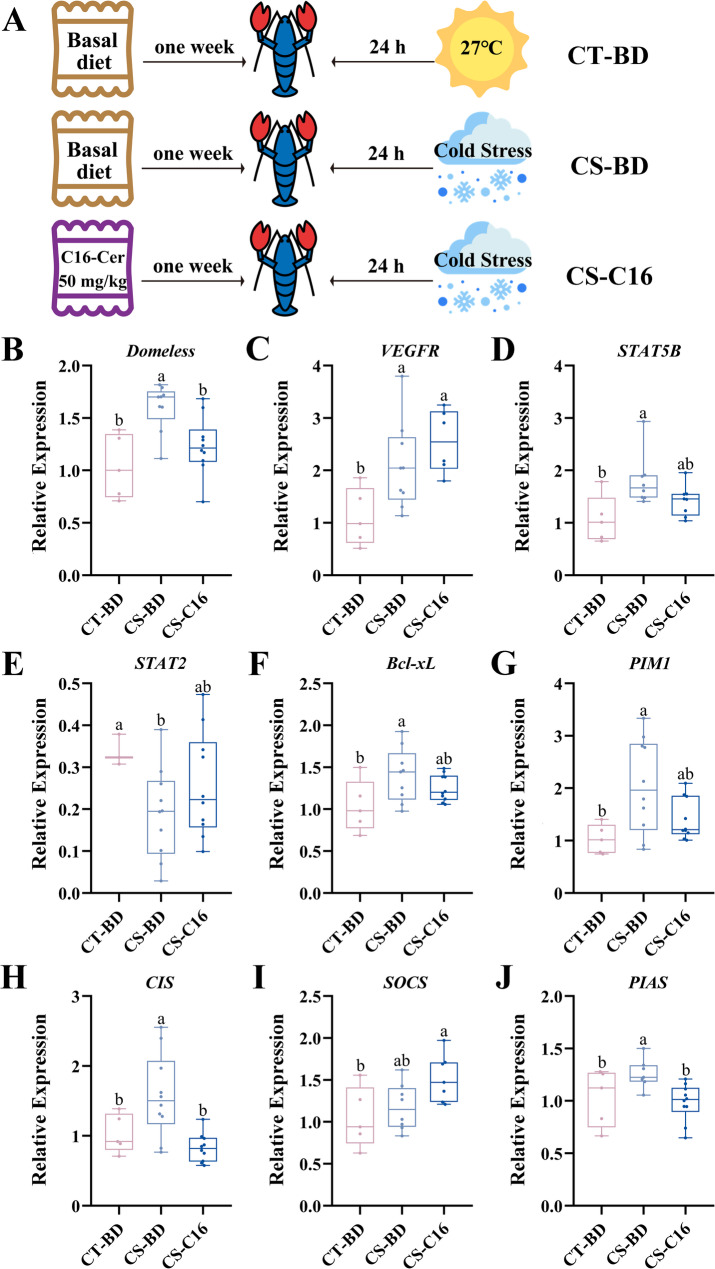
Modulation of JAK-STAT pathway by dietary C16-ceramide under cold stress in male *C. quadricarinatus*. (**A**) C16-ceramide induces comprehensive pathway suppression in male *C. quadricarinatus*. The expression of *VEGFR* (**D**), *STAT5B* (**E**), *STAT2* (**F**), *Bcl-xL* (**G**), *PIM1* (**H**), *CIS* (**I**), *SOCS* (**J**), and *PIAS* (**K**) of JAK-STAT pathway were analyzed across three experimental groups: ambient-temperature (27 °C) with basal diet (CT-BD), cold-stress with basal diet (CS-BD), and cold-stress with C16-ceramide diet (CS-C16). Data presented as mean ± SD (*N* = 6 per group)

Conversely, in males, C16-ceramide selectively modulated pathway components without global suppression (Fig. 7). While *Domeless* induction was partially attenuated, *VEGFR* remained fully activated (Fig. 7A and B). Crucially, C16-ceramide rescued inflammatory signaling by promote the expression of *STAT2* in CS-C16 group compare with CS-BD group, and maintained the expression of *STAT5B* in CS-C16 as CS-BD group (Fig. 7D and E). Survival effectors *Bcl-xL* and *PIM1* subjected to a minor impact but remained induced (Fig. 7F and G), negative feedback in CS-C16 group was potentiated through *SOCS* overexpression, while *PIAS* and *CIS* was suppressed (Fig. 7H, I and J).

### C16-ceramide modulates antioxidant defense in a sex specific manner

**Fig. 8:**
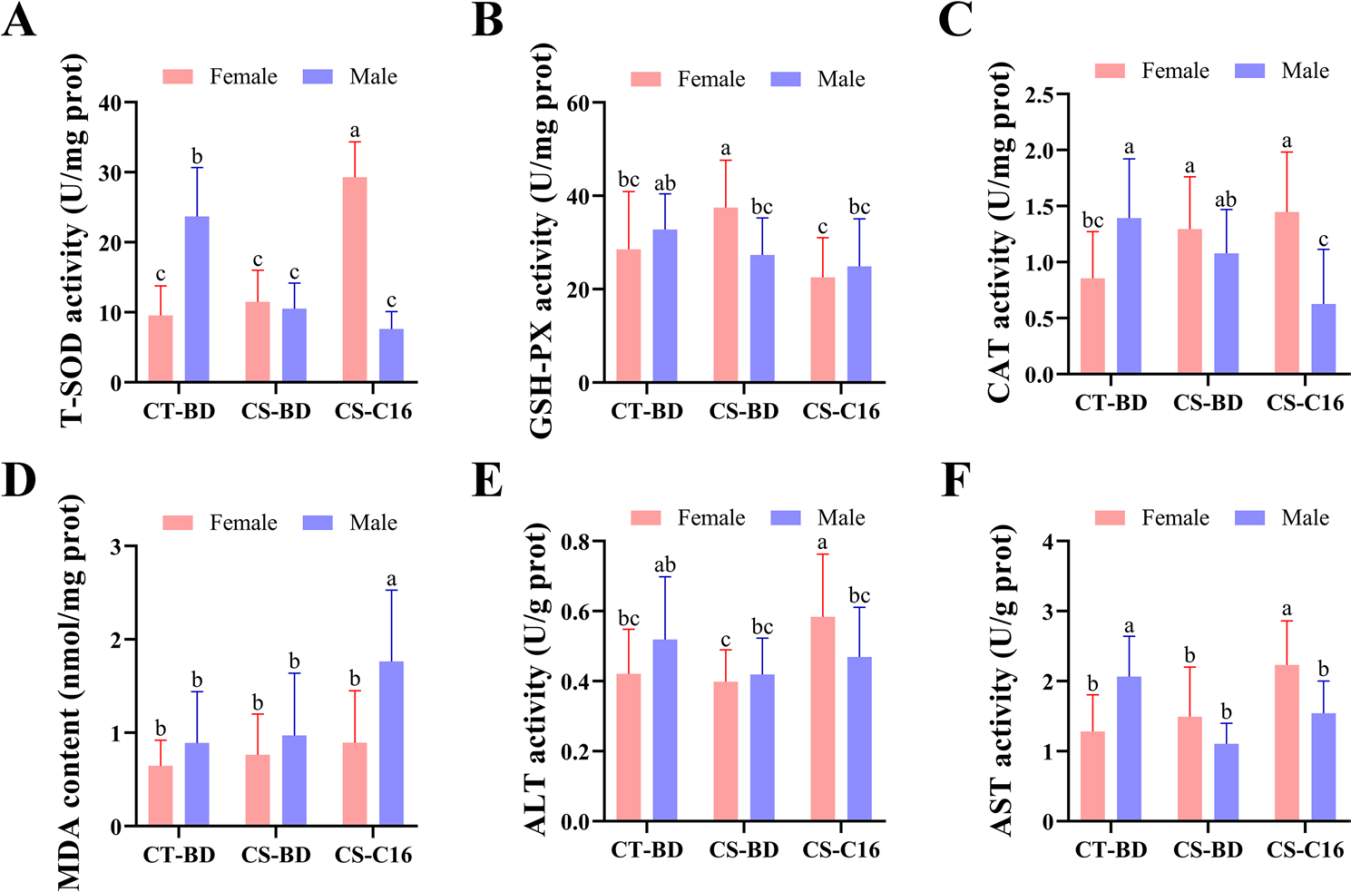
Analysis of oxidative stress markers in *C. quadricarinatus* under cold stress and dietary C16-ceramide supplementation. Expression levels of key oxidative stress markers were measured in hepatopancreas samples across three groups: Ambient-temperature basal diet (CT-BD), cold-stress basal diet (CS-BD), cold-stress C16-ceramide diet (CS-C16). Panels show T-SOD activity (**A**), GSH-PX activity (**B**), CAT activity (**C**), MDA content (**D**), ALT activity (**E**) and AST activity (**F**). Pink bars represent female group (48 h cold exposure), blue bars represent male group (24 h cold exposure). Data represent mean values ± SD

C16-ceramide supplementation elicited profoundly divergent effects on oxidative stress responses between male and female *C. quadricarinatus* during cold exposure (Fig. 8). The baseline comparisons revealed males possessed constitutively higher T-SOD, CAT and AST activities (Fig. 8A, C and F), while maintaining comparable GSH-PX, MDA, and ALT levels (Fig. 8B, D and E). In females, C16-ceramide significantly enhanced T-SOD activity, while maintaining CAT at cold stress induced levels. However, it suppressed GSH-PX activity and elevated ALT and AST levels. Conversely, in males, C16-ceramide significant suppressed the CAT activity and markedly increased MDA content. Unlike females, GSH-PX, ALT and AST levels remained unaltered in males.

## Discussion

This study reveals distinct male and female strategies for coping with cold stress in red claw crayfish (*C. quadricarinatus*), driven by molecular differences identified through transcriptomic and metabolomic analyses. Although the number of DEGs was comparable between sexes, their functions differed significantly, pointing to sex-specific defenses shaped by different physiological needs.

Males showed a natural advantage in handling oxidative stress, with higher baseline levels of key antioxidant enzymes (AST, T-SOD, CAT). Enhanced BCAA catabolism and nucleotide metabolism for quick energy [36], and ABC transporter activity was increased to maintain cell stability [37]. Importantly, activity in the energy-intensive PI3K-Akt pathway [38, 39] was reduced, likely to focus resources on essential defenses. This setup allows males to tolerate the brief burst of damaging molecules (oxidative burst) that happens when they quickly activate the JAK-STAT pathway. This fast response, higher risk strategy suits their ecological functions in competing for mate competition and defending territory [40, 41]. Females, in contrast, prioritized long-term activation of the JAK-STAT pathway to maintain overall metabolic balance. This conservative strategy minimizes oxidative damage, crucial for protecting their investment in reproduction (like egg development). They relied more on changing gene activity rather than major metabolic shifts to preserve ovarian resources.

The temporal dynamics of JAK-STAT activation further reflect this dichotomy. Males exhibit rapid-transient induction (Fig. 3) for immediate survival, whereas females sustain signaling (Fig. 4) for systemic reprogramming. This parallels tissue-specific *STAT* modulation in hibernating mammals [42], illustrating how conserved pathways enable diverse adaptive outcomes across biological scales—from tissue-level adjustments in vertebrates to sex-dimorphic strategies in invertebrates.

Evolutionarily, the observed divergence in cold adaptation strategies aligns with the evolutionary principle of trade-offs between reproduction and survival driven by differential reproductive investment between sexes. In females, the sustained JAK-STAT activation prioritizes metabolic homeostasis and long-term transcriptional reprogramming, which is a conservative strategy minimizing oxidative damage to protect reproductive resources. This is consistent with their higher energetic investment in reproduction, as evidenced by elevated vitellogenin synthesis costs in crustaceans [22, 43]. Conversely, males employ a rapid, antioxidant-dependent response that take more risks by accepting transient oxidative stress to ensure immediate survival, align with their higher basal metabolic rates and activity demands [44, 45]. These sex-dependent variations in adaptive homeostasis are conserved across species, primarily driven by hormonal exposure [46], and mating behaviors [47]. This fundamental trade-off between reproductive resource preservation and survival agility reflects a conserved pattern across vertebrates and invertebrates [48, 49]. For instance, female *Oncorhynchus mykiss* often exhibit lower cortisol concentrations in ovarian fluid and oocytes to protect ovarian function under stress [50], while male fish prioritize rapid ATP generation for aggressive contest [51].

Sphingolipid metabolism, is important for cell survival across species [52–54]. However, our findings reveal its adaptive repurposing for environmental stress responses, consistent with the important role in low-temperature resistance in the cold-tolerant strains of *Litopenaeus vannamei* [55]. The sphingolipid hub molecule C16-ceramide [56] was an important factor in this reproductive and survival trade-off, which functions as a sex-dimorphic metabolic brake. Its coordinated downregulation in both sexes modulates defense strategies with differential costs. In females, C16-ceramide potently suppresses JAK-STAT signaling, exhibit broad pathway inhibition and CIS upregulation to prevent immunometabolic hyperactivity, but at the cost of hepatopancreatic injury (elevated ALT/AST, suppressed GSH-Px). In males, high basal antioxidant capacity mitigates the inhibitory effects of C16-ceramide on JAK-STAT (minimal pathway disruption), but its pro-oxidant properties still reduce CAT activity and trigger MDA accumulation [57]. Specifically, in females, it prioritizes metabolic conservation by preventing JAK-STAT over-suppression, while in males, it permits tolerance of controlled oxidative risk within their elevated antioxidant capacity.

The functional plasticity of the JAK-STAT signaling pathway under the regulation of this sphingolipid metabolism exists in human cells as well. The representative molecule, ceramide, can activate the JAK2-STAT1/3 pathway in normal cells to drive pro-inflammatory responses [58], and can selectively inhibit the JAK1-STAT3 pathway in cancer cells to exert anti-tumor effects [59]. This plasticity feature reveals that the functional output of JAK-STAT under the regulation of ceramide is context dependent. Future studies should address the direct regulation of C16-ceramide during *STAT* phosphorylation and its crosstalk with antioxidant enzymes to fully elucidate this sex-specific regulatory axis.

## Conclusions

In summary, our integrated transcriptomic and metabolomic analysis reveals sex-specific molecular adaptation strategies in *C. quadricarinatus* eyestalk tissue under cold stress. We demonstrate that females activate a JAK-STAT centered signaling network enabling continuous transcriptional reprogramming, while males exhibit metabolic remodeling and enhanced basal antioxidant capacity. Both sexes conserved energy by downregulating high-energy processes including oxidative phosphorylation and sphingolipid signaling. Coordinated downregulation of C16-ceramide across sexes represents an adaptive strategy to prevent sex-specific toxicity. In females, it avoids suppression of JAK-STAT components and antioxidant imbalance, in males, it prevents severe disruption of antioxidant homeostasis. These findings provide molecular evidence for sexual dimorphism in crustacean cold adaptation, indicating C16-ceramide’s differential regulatory role in sex-specific cold resistance mechanisms.

## Supplementary Information

Supplementary Material 1.

## Data Availability

The datasets used and/or analyzed during the current study are available from the corresponding author on reasonable request.
