# Supplementary material for: C16-ceramide modulates sex-dimorphic defense strategies under cold stress in Cherax quadricarinatus via JAK-STAT suppression and oxidative disruption
Source: Biol Sex Differ. 2026 Mar 26;17:97. doi: 10.1186/s13293-026-00892-y (PMC13147606; doi:10.1186/s13293-026-00892-y)
Supplement: Supplementary file 1 — Supplementary Material 1. [file 13293_2026_892_MOESM1_ESM.docx]

Supplementary Table 1 Primer sequences used for transcriptomic data validation in *Cherax quadricarinatus*

| Gene name | Primer name | Sequences (5’-3’) |
| --- | --- | --- |
| *ALF* | Forward | GGAGAAGCGACCACCAGAAG |
|  | Reverse | CCACTGCTGGGCTGATTGTT |
| *HSP70* | Forward | GTGGTATGCCCGGTGGTATG |
|  | Reverse | GGTAGGACCAGAACCACCAC |
| *VOM1* | Forward | TTGGAGAATGGACCGGCAAA |
|  | Reverse | GGGTCTGTATGCCGCAGATT |
| *Trx* | Forward | AGTGTTTGTGGGAAGCGTCT |
|  | Reverse | CCGTACTGTCCTCCCAAACC |
| *COX2* | Forward | TATGCTACCTTCGCACGGTC |
|  | Reverse | AAGGAATTCGGCAAAAGGGC |
| *CAT* | Forward | TGATCGAGAACGCATCCCAG |
|  | Reverse | GCCACTCTCACCACCTACAG |
| *18S rRNA* | Forward | CTGAGAAACGGCTACCACATC |
|  | Reverse | GCCGGGAGTGGGTAATTT |


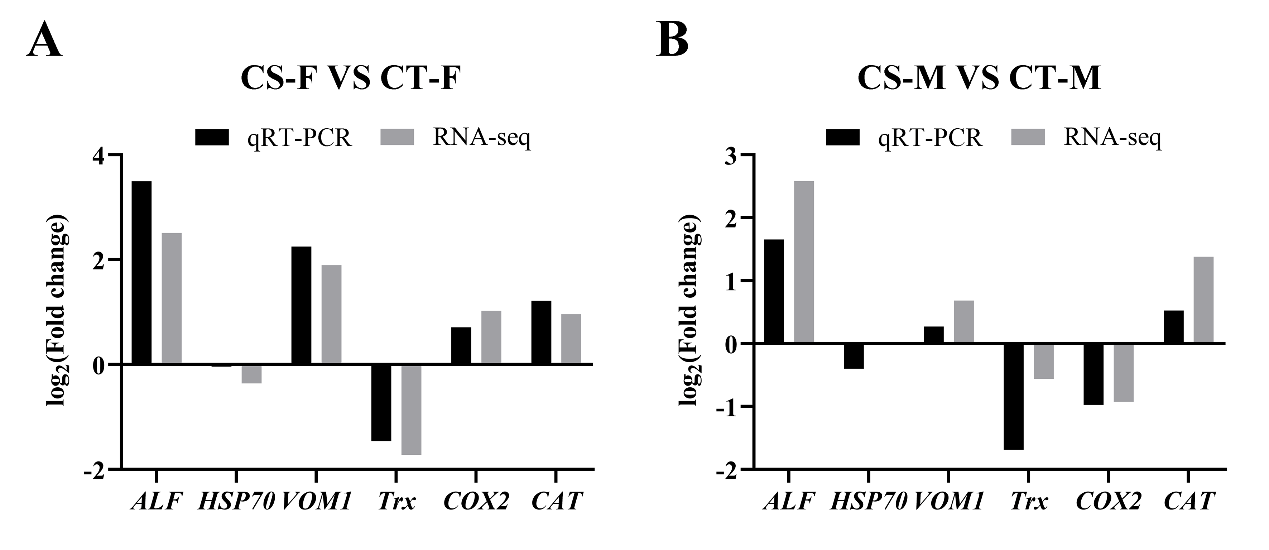


Supplementary Figure 1 Validation of transcriptomic data by qRT-PCR.

Note: Expression levels of six candidate genes (*ALF*, *HSP70*, *VOM1*, *Trx*, *COX2*, *CAT*) in cold-stressed versus control groups in female (A) and male (B) group. Data were normalized to 18S rRNA and analyzed using the 2^−ΔΔCT^ method. Pearson’s correlation coefficient (R-value) was 0.97 and 0.95, which showed a high consistency of the RT-PCR and RNA-seq data. Reactions were performed in 5 biological replicates and 3 technical replicates.


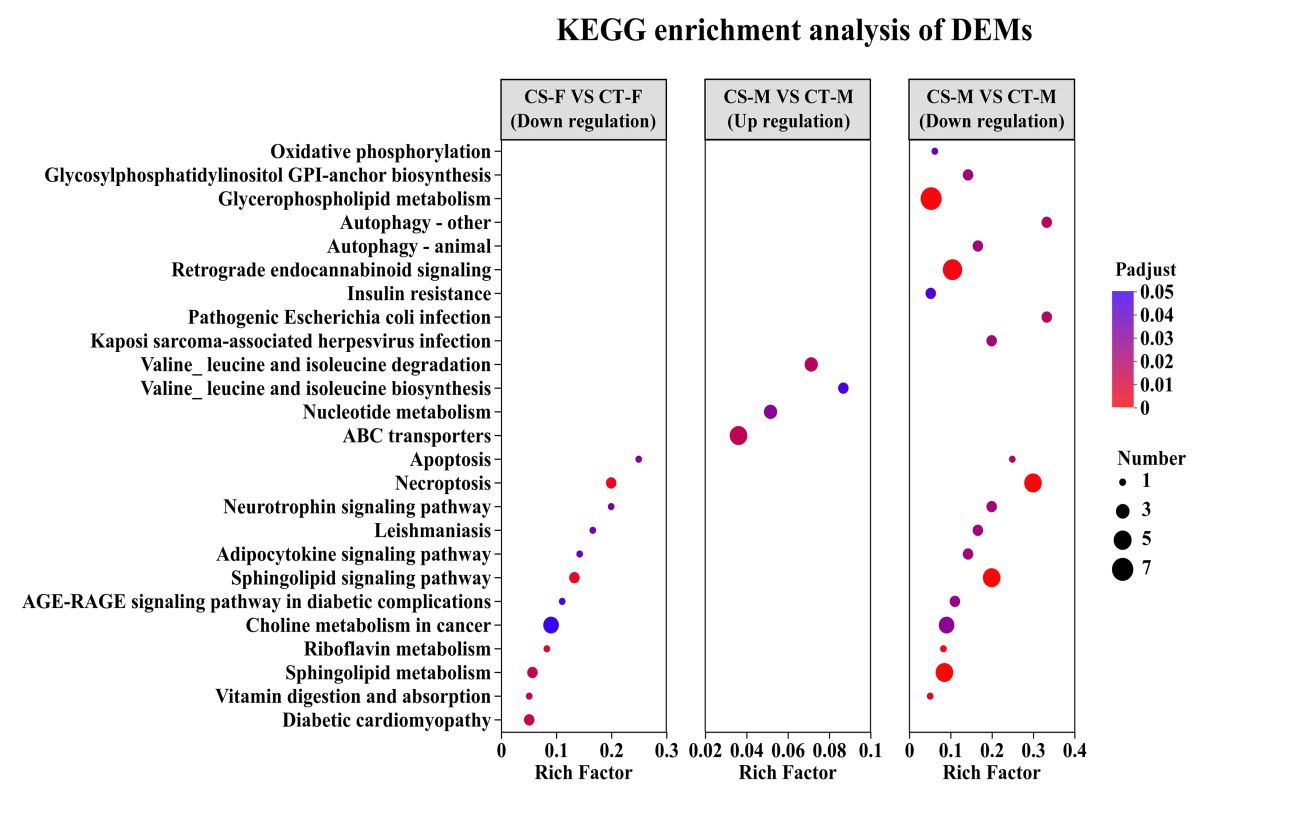


Supplementary Figure 2 KEGG pathway enrichment analysis of DEMs.

Note: Bubble plots display the KEGG enrichment results for three subsets of DEMs identified between CS (cold stress treatment group) and CT (control group) comparison. No significant pathways were enriched by DEMs with higher expression in CS female group vs CT female group. The top 20 most significant pathways are displayed.
